# Adult survival has a stronger role than productivity in the annual population change of European songbirds

**DOI:** 10.1007/s00442-025-05810-4

**Published:** 2025-10-11

**Authors:** Inari Nousiainen, Laura Bosco, Petteri Lehikoinen, Rob Robinson, Juan Arizaga, Jaroslav Cepák, Wolfgang Fiedler, Olaf Geiter, Ian Henshaw, Christof Herrmann, Marc Illa, Henk P. van der Jeugd, Bert Meister, Arantza Leal, Péter Lovászi, Simone Pirrello, Markus Piha, Aleksi Lehikoinen

**Affiliations:** 1 LUOMUS—Finnish Museum of Natural History, University of Helsinki, PL 17 - P.O. Box 17, 00014 Helsinki, Finland; 2 British Trust for Ornithology, The Nunnery, Thetford, Norfolk, IP24 2PU UK; 3 Aranzadi Ringing Scheme, Aranzadi Sciences Society, Zorroagaina 11, S20014 San Sebastián, Spain; 4 Bird Ringing Centre, National Museum, Hornoměcholupská 34, 10200 Prague 10, Czechia; 5 Max Planck Institute of Animal Behavior Centre for Animal Marking Am Obstberg 1, 78315 Radolfzell, Germany; 6 Institute of Avian Research, An der Vogelwarte 21, 26386 Wilhelmshaven, Niedersachsen, Germany; 7 Swedish Bird Ringing Centre, Department for Nature and Environmental Monitoring, The Swedish Museum of Natural History, Box 50007, 104 05 Stockholm, Sweden; 8 Agency for Environment, Nature Conservation, and Geology Mecklenburg-Western Pomerania, Hiddensee Bird Ringing Scheme, Goldberger Str. 12B, E18273 Güstrow, Germany; 9 ICO-MUS. NAT. SCI BARCELONA (ESC), Institut Català d’Ornitologia, Nat-Museu de Ciències Naturals de Barcelona, Plaça Leonardo da Vinci 4-5, 08019 Barcelona, Spain; 10 Dutch Centre for Avian Migration and Demography, Droevendaalsesteeg 10, 6708 PB Wageningen, The Netherlands; 11 Bonhoefferstr. 5, E04668 Grimma, Germany; 12 Paser, SEO/BirdLife, C/Melquiades Biencinto 34, 28053 Madrid, Spain; 13 MME/BirdLife Hungary, Költő U. 21., 1121 Budapest, Hungary; 14 Area Avifauna Migratrice (BIO-AVM), Istituto Superiore Per La Protezione E La Ricerca Ambientale (ISPRA), Via Ca’ Fornacetta 9, 40064 Ozzano Dell’Emilia, BO Italy; 15 LUKE—Natural Resources Institute Finland, Latokartanonkaari 9, 00790 Helsinki, Finland

**Keywords:** Demography, Productivity, Survival rates, Population change, Passerines

## Abstract

Biodiversity is decreasing at an alarming rate, and there is an urgent need to understand the demographic drivers behind population declines. Therefore, it is important to study the different stages of a species’ life cycle, including adult survival and productivity. It is still poorly understood whether adult survival or productivity has a stronger role in population change, and how the role of adult survival and productivity varies spatially in relation to species’ traits. We used bird ringing data from the European Constant Effort Sites (EuroCES) project from the years 2000 to 2021, with 1.2 million captures of 33 songbird species from ten European countries. We investigated the role of productivity and adult survival in annual population change and how it was affected by spatio-climatic gradient (measured as average breeding season temperature per country), migratory strategy (long- vs. short-distance and sedentary birds), and breeding habitat (forest vs. reeds) using linear mixed effect models. Overall, our results show that adult survival is a more important driver of annual population change than productivity within European songbirds. The importance of adult survival and productivity varied spatially, both having a weaker influence in warmer regions. Furthermore, the role of adult survival was stronger for long-distance migrants compared to short-distance migrants. Acknowledging that adult survival has a stronger role in annual population change than productivity can help design more robust conservation actions, especially regarding long-distance migratory songbirds.

**Supplementary Information:**

The online version contains supplementary material available at 10.1007/s00442-025-05810-4.

## Introduction

Earth's biodiversity is facing excessive anthropogenic pressures, which have led to declines and even extinctions in numerous species and populations (IPBES 2019). To mitigate the effects of these pressures, it is important to understand the underlying demographic drivers of population trends (e.g., Anderson et al. 2009; Selwood et al. 2015). A population can be influenced by demographic parameters, such as survival, productivity, immigration, and emigration, and among them, survival and productivity are often the easiest to measure (e.g., Begon and Townsend 2020). However, it is still widely unknown whether populations are more affected by survival or productivity (but see Sæther et al. 2016; Nater et al. 2023) and how the location of populations influences these demographic parameters, as population trends of species are known to differ within their range along the spatio-climatic gradient (Jiguet et al. 2010; Lehikoinen et al. 2016). Different demographic mechanisms may drive regional variations in population changes as populations situated on the cold edge of a species’ occurrence can be more limited by climatic variables, whereas populations on the warm edge can be more affected by species interactions (Pearce-Higgins and Green 2014; Paquette and Hargreaves 2021). Population change can also be strongly impacted by species traits, for example, through migratory behavior (e.g., Zylstra et al. 2022; Newton 2024) or habitat preference (e.g., Bowler et al. 2019; Reif and Hanzelka 2020). It is thus additionally important to understand how species’ demography and the importance of survival and productivity on population trends may be affected by species’ traits.

When studying demographics, birds are widely recognized as an excellent study group as their ecology is well known, there are high-quality, long-term datasets, and they react to environmental changes at reasonable spatial and temporal scales (Morrison 1986; EEA 2005; Gregory et al. 2005). Hence, bird demography has been a widely studied subject; yet, especially in the case of survival, such studies have been focusing mostly on single species or restricted geographic areas (e.g., Siriwardena et al. 1998; Bijlsma et al. 2012; Fay et al. 2020). However, in recent years, demographic studies with a broader, cross-continent, and multispecies view have been conducted using bird ringing and survey data (e.g., Johnston et al. 2016; Hanzelka et al. 2019, 2024; Morrison et al. 2021, 2022; Youngflesh et al. 2023). Johnston et al. (2016) demonstrated that there is a relationship between seasonal weather conditions, adult survival, and changes in abundance of eight species of Western European warblers. Furthermore, Hanzelka et al. (2019) analyzed how population trends of European birds are related to traits that mirror the influence of major environmental drivers and demonstrated that these drivers have different impacts across Europe, whereas Hanzelka et al. (2024) showed how climate influences the breeding productivity of long-distance migratory passerines of Europe and how their responses to different climatic variables were not linear. Additionally, in their studies of the demography of European breeding passerine birds, Morrison et al. (2021, 2022) showed that productivity varies more spatially while survival varies more between years and between species with different migratory strategies. In addition, the species breeding in the same areas tend to have similar trends in productivity independent of their migratory behavior. Similar studies have also been conducted in North America, where Youngflesh et al. (2023) demonstrated how asynchrony in breeding bird phenology has affected the productivity of 41 breeding North American bird species. However, as these studies either focus on long-term demographic trends or are limited in the spatial or temporal scale of their datasets, we are currently unaware of how survival and productivity influence annual population change on a continental scale. Especially linking these effects with the spatio-climatic gradient and species traits offers an important avenue for a deeper understanding of the drives of species demography.

In this study, we investigated how changes in adult survival and productivity influence the annual population change of European songbirds using a cross-continental, multispecies, and long-term dataset. We used bird ringing data from the European Constant Effort Sites (EuroCES) project (Robinson 2023), covering 33 species of songbirds, 1.2 million captures, and ten countries spanning from North Europe to the Mediterranean during the years 2000–2021. All our study species are small-bodied songbirds of which adults, despite being relatively short-lived, can live up to 5–15 years (Valkama et al. 2014), which makes them a good taxon to study annual population change and its demographic drivers. More specifically, we asked: (1) Are there differences between adult survival and productivity in explaining the annual population change of European songbirds? (2) Does the role of adult survival or productivity vary along the spatio-climatic gradient? (3) Is the role of adult survival or productivity affected by species traits such as migration strategy or breeding habitat? It is important to test if among songbirds there are differences between adult survival and productivity regarding their role for annual population change, as earlier studies have shown differences in their spatial and temporal roles in shaping bird species demographics (e.g., Morrison et al. 2021, 2022). Understanding these differences has important conservation implications as it provides knowledge on where and when to implement conservation actions for which species groups. We hypothesized that the role of adult survival and productivity in explaining annual population change varies along a spatial gradient, in accordance with earlier studies investigating long-term population trends (e.g., Jiguet et al. 2010; Lehikoinen et al. 2016). We further predicted that the role of adult survival or productivity in population change varies between species’ migratory strategy and breeding habitat, because of the different population change between migratory (e.g., Sanderson et al. 2006; Vickery et al. 2014; Howard et al. 2020) and breeding habitat species groups (e.g., Bowler et al. 2019; Reif and Hanzelka 2020). For instance, long-distance migrants typically have less time to breed than short-distance migrants or residents (Hedenström 2007), and long migration journeys increase adult mortality (Alerstam et al. 2003; Newton 2024). Hence, long-distance migrants likely have less possible variability in their productivity, whereas adult survival has more apparent potential to increase, and thus it may be more important for their population change. Furthermore, as there are differences in bird species trends regarding their main breeding habitats (e.g., PECBMS 2024), we predict that the role of adult survival and productivity on population change might differ between breeding habitats.

## Materials and methods

### Bird ringing data

The Constant Effort Site project (CES) follows a standardized bird ringing protocol, in which birds are captured with mist nets throughout the breeding season. When captured, birds are either marked by placing a metal ring with a unique alphanumeric code on their leg, or if a bird is already ringed, its code will be checked and recorded as a recapture. Additionally, the bird’s species, sex, and age are determined and noted. Most of the species captured with mist nets are small-bodied songbirds (EURING—CES in Europe 2023). Bird ringing in Europe is done by licensed, voluntary bird ringers, and it does not need additional ethical approval. The procedure to obtain a ringing license differs between countries, and they are coordinated by each country's ringing schemes individually (for scheme-specific information, see EURING—EURING Articles 2024).

A single CES season usually includes 7–12 mist-netting visits, which are done at regular intervals and aim to cover the whole breeding season while excluding the periods of peak passage. The trapping is typically done in the early morning hours for the same length of time between years. The length and timing of the trapping season differ between latitudes and range from April to August. Typically, CES sites are situated in scrub, reedbeds, or deciduous woodlands. While the number and position of mist nets differ between sites, they mainly remain the same on a given site between years. We obtained our data in February 2023 through EURING, the European Union for Bird Ringing, which gathers and coordinates CES data from national ringing schemes in a European-level project called EuroCES (Robinson et al. 2023). Overall, the data consist of 2070 CES sites within 19 ringing schemes, expanding across 15 European countries, including 309 bird species and 4.5 million captures since the 1980s.

### Data selection

We limited our analysis to the years 2000–2021, as only a few national CES schemes were established before that period, and our data did not expand beyond the year 2021. We chose only those schemes where most of the sites had been visited 7–12 times per year, to make the schemes more comparable. Every chosen scheme had its maximum visit number, which was used for site selection, and was defined by the modal number of visits that were done during a year within that scheme’s sites, throughout all the years the scheme had been active. If a scheme had periods of different activity, the years with higher maximum visit numbers were selected. Chosen years among schemes ranged from 7 to 22 (for scheme-specific values, see Supplementary material, Table S1).

For six of the schemes, the maximum visit number was 12, but for five of the schemes, it ranged from seven to 10. We combined different schemes for the analysis within a country, except in Spain, since the methodology of the four Spanish schemes differed (from the four schemes, two were combined, resulting in three Spanish schemes in the analysis; for details, see Supplementary material, Table S1). In addition, two countries were considered as one scheme in the analysis as the Republic of Ireland is participating in the British ringing scheme. For the scheme-specific site selection, we followed the criteria of Morrison et al. (2022) with small modifications:

Our analysis included only sites that

1. had been running for five or more years, and from those years, only the years that
2. were visited at least 2/3 times of the maximum visit number per year, including at least 1/4 times of the maximum visit number in each of the first and second halves of the breeding season, and,
3. for each species, at least two adults (older than first calendar-year birds) and two young (first calendar-year birds) were captured every year.

**Fig. 1:**
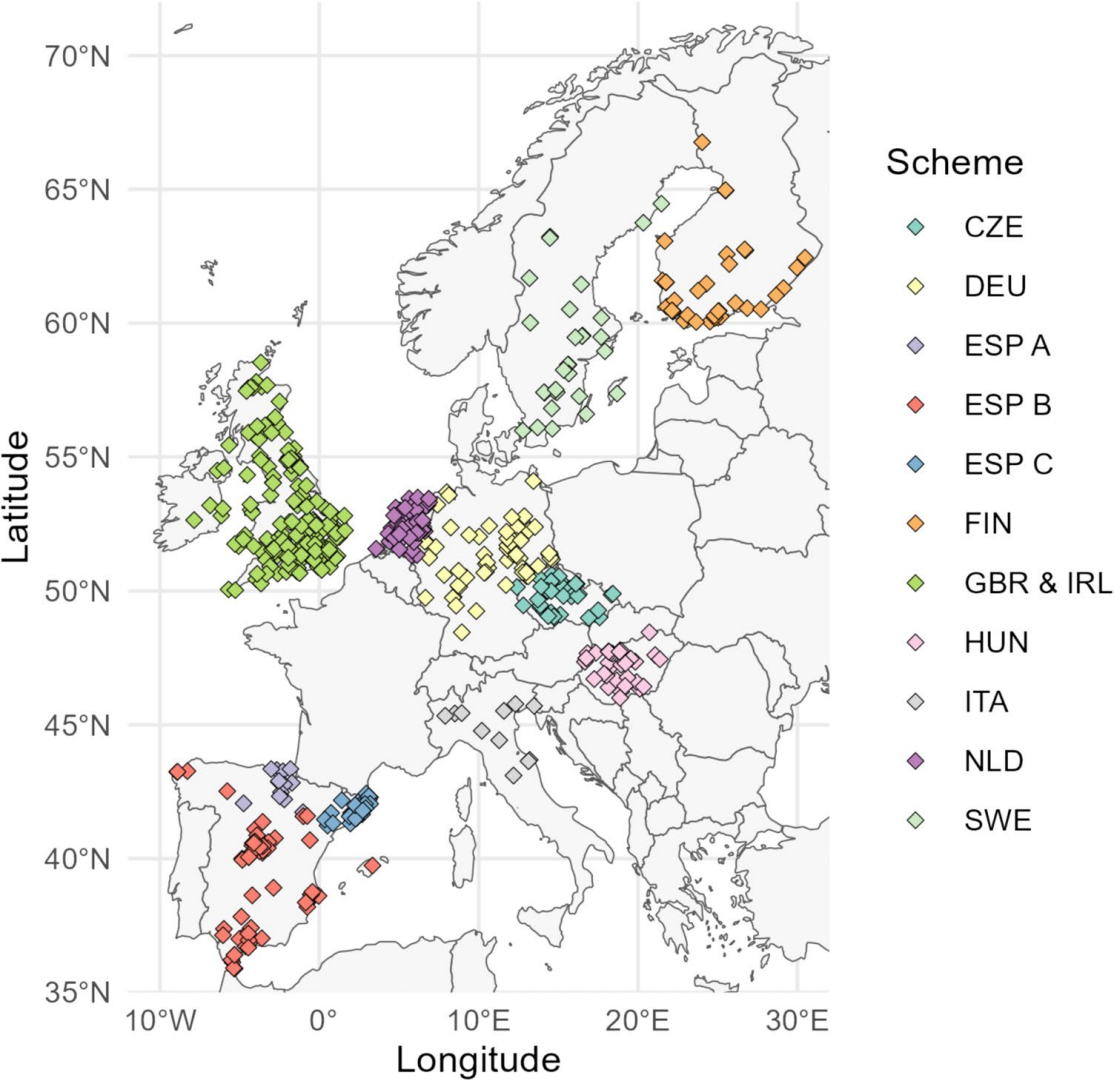
Map of the spatial distribution of the constant effort ringing sites (CES) chosen for the analysis, colored by the ringing scheme. Acronyms of countries belonging to the same scheme are listed. The three schemes of Spain are separated with letters A, B, and C. CZE = Czechia, DEU = Germany, ESP A = Spain Aranzadi, ESP B = Spain, ESP C = Spain Catalonia, FIN = Finland, GBR = Britain, IRL = Republic of Ireland, HUN = Hungary, ITA = Italy, NLD = Netherlands, SWE = Sweden

In the end, ten countries, 11 schemes, and 583 sites were selected for the productivity analysis, and 562 sites were selected for the survival analysis. The number of sites selected varied between species as the third criterion mentioned above excluded sites only at the species-specific level, i.e., not every site had to include all the study species included in our dataset. The excluded countries, schemes, and sites were those with a maximum visit number of less than seven or with sites unmatching our selection criteria (for the selected schemes and sites, see Supplementary material, Table S1; for the scheme-specific numbers of sites before and after applying selection criteria in productivity and survival analysis, see Supplementary material, Figs. S2 and S3; for the map of the chosen sites, see Fig. 1).

For species selection, we included in the analyses only those species within a scheme that had on average at least 50 individuals captured per year (after DeSante et al. 2009) from all sites and all years of the scheme during the period 2000–2021. Overall, 33 species of passerines were selected, ranging from 3 to 21 species per scheme (for the selected species, see Supplementary material, Table S2; for the scheme-specific numbers of species before and after applying selection criteria, see Supplementary material, Fig. S4). The final number of species and sites also depended on the criteria used later in the analyses (see data analyses for details). All the species were small-bodied passerines with relatively short generation times as they reached the breeding age already in their second calendar year; therefore, they are considered young only in their first calendar year.

The threshold to include only species that had on average 50 trapped individuals per year was compared to other threshold values in the sensitivity analysis (see below).

### Data of spatio-climatic gradient, species’ traits, and phylogeny

To study the potential differences in the responses of species along a spatio-climatic gradient, we used the country-specific mean breeding season temperature across the years 2000–2021 as a proxy. Temperature data were extracted as a gridded monthly average temperature from April to August from the CRU TS dataset at 0.5° spatial resolution (Harris et al. 2020) and averaged across the entire study period. In the analysis, country-specific means were treated as scheme-specific, as for most of the countries, there was only one scheme. For all three Spanish schemes, we used the average temperature of Spain as the temperature differences inside the country were minimal. For the scheme combining Britain and the Republic of Ireland, we used temperature data from Britain as the majority of sites were situated there. We chose the average temperature as it captures both latitudinal and longitudinal differences in the European climatic conditions.

For species traits, we used migratory strategy and breeding habitat. For both traits, we divided the species into two groups following the trait information obtained from the AVONET database (Tobias et al. 2022). For migratory strategies, we distinguished between the group of long-distance migrants (where the majority of the populations within a species’ range are long-distance migrants; *n* = 14 species; 48 populations from 11 schemes) and the group of short-distance migrants and sedentary birds (where all the populations are short-distance migrants or resident birds; *n* = 19 species; 72 populations).

For breeding habitats, the first group represented forest breeding birds (where at least part of the population breeds in forest habitat, *n* = 25 species; 96 populations) and the second group included birds that mostly breed in reed habitats (*n* = 8 species; 24 populations). As all species in the second group except for one species (*Sylvia melanocephala*, a scrubland breeding bird) breed in reed habitats, the second group is referred to hereafter as reed breeders.

As species with similar ancestors may show similar responses in their population change and trait relationships, we obtained species phylogeny data from the BirdTree.org database (Jetz et al. 2012) to account for the relatedness of species in a sensitivity analysis (see below).

### Data analysis

Our analyses were done in three steps. First, we used the constant effort ringing site data to calculate the annual adult survival, productivity, and population abundance (number of adults in the population) estimates for each species and scheme. We merged the site-level data per scheme when calculating the three estimates mentioned above because the uncertainty of the estimates would be higher in local estimates due to smaller sample sizes. Second, we used these values obtained in the first step to estimate the coefficients for how annual changes in population abundance are explained by changes in adult survival and productivity across all years for each species and schemes using a linear model. Third, we used the coefficients from step two to answer our three study questions using linear mixed models (for the workflow, see Supplementary Material, Fig. S1).

Step 1—For the first step of the analyses, we used the Cesr-package (Robinson 2023) in R (R Core Team 2022) to calculate the annual adult survival, productivity, and population abundance for each species in each scheme (Supplementary Material, Fig. S1, step 1). The package has been built for analyzing CES data specifically (Robinson 2023), and it has been used before to analyze demographic drivers (Arizaga et al. 2023). For the survival analysis (function mark.ces), the package uses the MARK program (White and Burnham 1999) through the Rmark-package (Laake 2013) and fits a modified CJS model (Cormack-Jolly-Seber; Lebreton et al. 1992), which also accounts for transient birds. From MARK, we gained two values: estimates for adult survival and estimates for recapture probability. In the model, survival is estimated annually but is assumed to be the same across all sites within a scheme. Recapture probability is the probability of the marked bird being captured again at the site. In the model, recapture probability is estimated at the site level through all years; therefore, we decided to use it to filter out the sites with unrealistically low or high recapture values (< 0.10 or > 0.90) for species-specific analysis. Overall, only capture histories of adult birds (older than the first calendar year) were used in the survival analysis. Adult survival was estimated for 427,227 individuals with 58,743 recapture occasions.

In addition to this, the MARK program provides annual standard errors (SE) for each species for all survival estimates (value between 0 and 1). In some cases, the SEs of the annual survival estimates were very high or very low, making the estimates unreliable. We thus decided to use only those years where SEs of survival estimates were smaller than 0.25 but over 0.01 to ensure that survival estimates were robust. This was done at the species and scheme level; therefore, excluding a year from one species in a specific country did not influence the other species or schemes. Overall, unreliable SE values were found for 334 scheme–species–year combinations, resulting in a final number of 1,890 observations for each of our three parameters (adult survival, productivity, and adult abundance).

Productivity and population abundance are modeled in Cesr by fitting a generalized linear model (GLM) with a logit-link function. For productivity, Cesr uses an event-trial framework as a response variable, where the trial is one capture, and the ‘success’ is one capture of a young bird. Explanatory variables are the site and the year, while the error distribution is quasi-binomial. Adult abundance is modeled by the number of individuals captured per site and year as a response variable. Explanatory variables are site and the year, while the error distribution is quasi-Poisson (function index, for details, see Robinson 2023). Through fitting the GLM models, we obtained a dispersion parameter for all productivity and adult abundance estimates, where less than four is a sign of good model fit (after Burnham and Anderson 2002), and we thus only included those species for which the dispersion parameter was under four. In the productivity analyses, both the captures of adult (older than the first calendar year) and young (first calendar year) birds were used, while in adult abundance estimates, only captures of adult birds were used. Adult abundance was estimated for 485,970 individuals, whereas productivity was estimated for the same number of adult individuals, alongside 712,823 juveniles.

Finally, we excluded all those species where the Cesr was not able to calculate estimates for the indices for all the years, resulting in the final number of 33 species used in analyses (for the species- and scheme-specific numbers of individuals in the survival analysis, see Supplementary material, Table S3; for the species- and scheme-specific numbers of adults and young birds in productivity and population abundance analysis, see Supplementary material, Tables S4 and S5).

Step 2—For the second step of the analysis, we calculated the annual population change as the growth rate in the population abundance using the annual estimates of population abundance from step one (Fig. S1): $r=\text{log}(1+\frac{{x}_{i+1}-{x}_{i}}{{x}_{i}})$

where *r* is the annual population growth rate, *x* is the adult abundance per year–species–scheme combination, and *i* refers to the respective year. We then ran a simple linear model, with the response variable being the annual change in population abundance from year x to year x + 1, and the response variables were annual adult survival (year x to x + 1) and productivity (year x; both obtained in step 1, Supplementary Material, Fig. S1). Annual estimates for adult survival and productivity were obtained in step one (Fig. S1). As the adult survival and productivity estimates are on different scales, we scaled them before modeling to make them more comparable. Using the scale function in R, both were scaled so that they had a mean of zero and a standard deviation of one. To take the different levels of uncertainty in our estimates into account, we used weights in all our models to adjust the influence each value in the response variable should have in the analysis based on its uncertainty. As model weight, we used the standard errors of adult survival, productivity, and adult abundance estimates produced by cesr (step 1, Fig. S1). We merged the standard errors of the three variables by taking their mean value per year, species, and scheme and used the inverse square of this averaged standard error (i.e., weight = 1/mean(SE)^2^). As a result, those year–species–scheme values with lower uncertainty in their predicted parameters had more power in the analyses. This model in step two (Fig. S1) produced coefficients for survival and productivity (*n* = 240), explaining the variation in population change for each species in each scheme across all years.

Step 3—In the third step, we built three final linear mixed models (LMMs) with a Gaussian error distribution to answer our study questions. In the models, the response variable was the coefficient of either survival or productivity from step two (lm-model estimates, step 2 in Fig. S1). This means that the species- and scheme-specific coefficients of survival and productivity were combined into one dataset, where each row corresponded to either the survival or productivity coefficient. The basic structure of the final models was: coefficient of demographic measure ~ demographic measure type + random effects + model weights (for detailed model structure, see Supplementary Material, Table S10).

To run the models, we used the functions lmer and lmerTest from the R package lme4 (Bates et al. 2015; Kuznetsova et al. 2017). To answer our first study question, whether adult survival or productivity was more important in explaining population change, the type of demographic measure (categorical variable: productivity or survival) was included as the explanatory variable, and scheme and species were set as random effects (model A). To answer our second study question, on the influence of the spatio-climatic gradient (continuous variable), we added an interaction between the demographic measure and average temperature per country to capture potential spatial differences (model B). Here, only the species was included as a random factor in the model as temperatures were already scheme-specific. To answer our third question about the role of species traits, we included the interaction between the type of demographic measure and migratory strategy (categorical variable: long-distance migrant vs. short-distance or resident species) and the type of demographic measure and breeding habitat (categorical variable: forest or reed habitats), with species and scheme included as random effect variables (model C). In all three models, the uncertainty of the survival and productivity coefficients was taken into account using their standard errors estimated in the model from step two as model weights (i.e., weight = 1/SE^2^). As a result, those coefficients with a lower uncertainty had more power in the analyses. We checked that the tested variables were not collinear using the Pearson correlation coefficient, with no strong pairwise correlations found (see Supplementary Material, Fig. S5).

Last, we performed four types of sensitivity analyses to test the robustness of our results related to data selection criteria, spatial, and phylogenetic autocorrelation. First, we tested the robustness of our selection criteria by comparing the results of three different selection criteria for study species: the strict criteria, moderate criteria (our chosen criteria above), and relaxed criteria. The results of these three options are shown in the Supplementary material, Table S6. Second, we tested our survival estimates filtering criteria by comparing the results from three different upper limits for the SE filter: 0.20, 0.25 (our chosen upper limit), and 0.30. The results of these three options are shown in the Supplementary material, Table S7. Third, we tested the spatial autocorrelation of the residuals using Moran’s I test through the testSpatialAutocorrelation—function from the DHARMa—package in R (Hartig 2024). The results of this last sensitivity analysis are shown in the Supplementary material, Table S8.

Fourth, we controlled for the phylogeny in the three above-mentioned LMM analyses (step three) by fitting phylogenetic generalized linear mixed models (PGLMMs) using the pglmm-function from the Phyr-package (Li et al. 2020)**.** However, the PGLMM was not able to include weights to account for the uncertainty in estimated indices and thus differed from the lmer model in this regard. Therefore, we also tested for a phylogenetic signal in the LMM model residuals from step three using the phylosig-function from the Phytools-package in R (Revell 2024) (for the results of phylogenetic analyses, see Supplementary material, Table S9).

## Results

### Importance of adult survival and productivity for population change

The results from calculating the estimates of adult survival, productivity, and adult abundance (step 1) and the coefficients of adult survival and productivity produced by the lm-model (step 2) are found in the Zenodo repository (see section Code Availability).

**Fig. 2:**
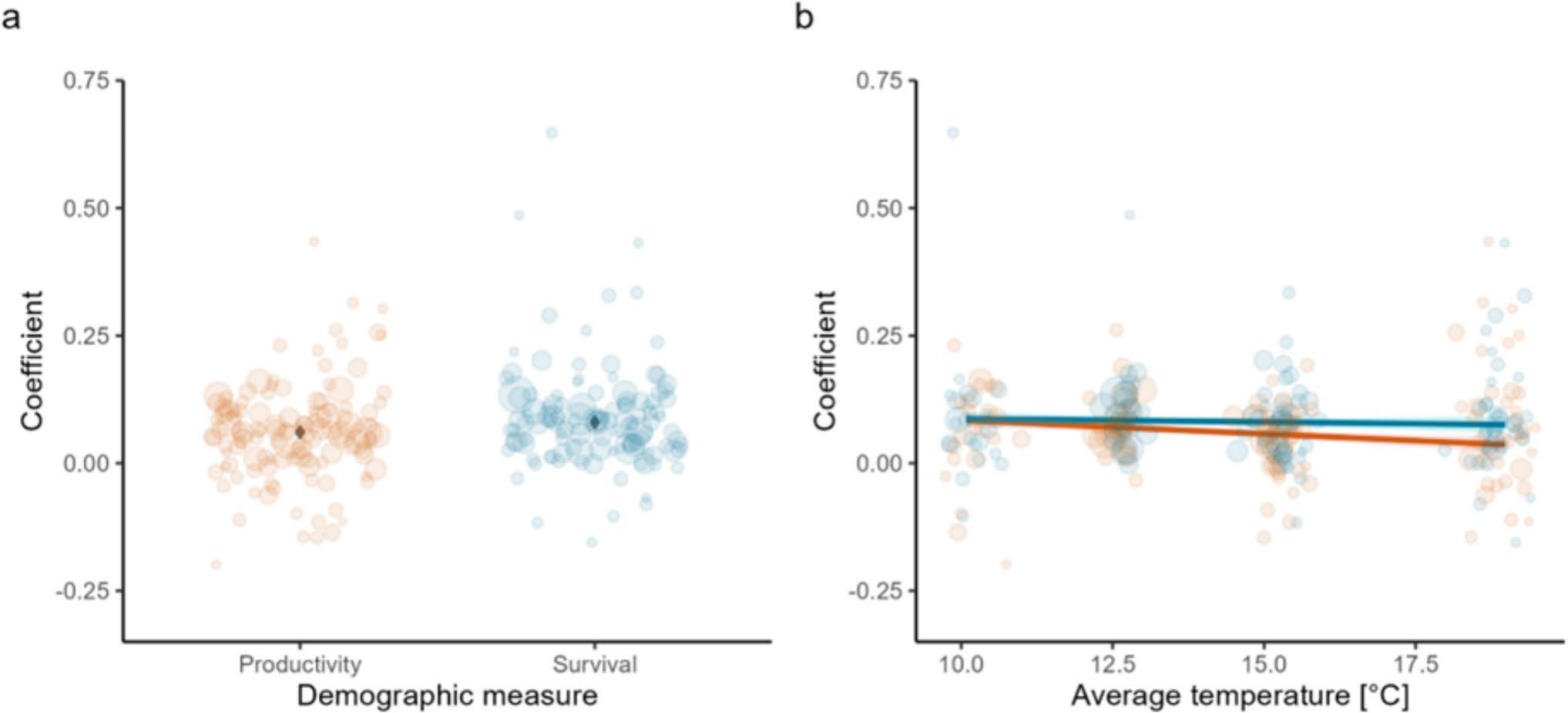
**a** The importance (coefficient) of productivity and survival on the population change of songbirds (*n* = 240, *P* = 0.010, model A in Table 1) and **b** how this connection varies along the spatio-climatic gradient (*n* = 240, *P* = 0.186 for the difference between productivity and survival in their response to average temperature of the country, model B in Table 1). The mean estimates and their confidence intervals are shown, along with the raw data points. The size of the datapoints indicates how much weight they have in the model, e.g., greater point size means a smaller standard error of the estimate and thus larger weight. Red = productivity, blue = adult survival

Model A—Significantly positive estimates for both the intercept (i.e., productivity) and slope coefficient (i.e., survival) suggest that both adult survival and productivity had a positive connection with the population change of the studied European passerines (Table 1A, Fig. 2a). However, because the slope coefficient, which represents the demographic measure of survival, had a significant effect, adult survival had a stronger contribution to the annual population change than productivity (productivity as a reference level; Table 1A, Fig. 2a).

**Table 1:** Parameter estimates and their standard errors, as well as test values of the LMMs explaining the role of survival, productivity, and other factors in the annual population change of songbirds

Parameter	Estimate ± SE	df	*t*	*P*
(A) Survival vs. productivity-model (*n* = 240, where n is species and country-specific coefficients)				
**Intercept**	**0.061 ± 0.007**	**25.310**	**9.290**	** < 0.001**
**Demog. measure (survival)**	**0.019 ± 0.007**	**218.600**	**2.612**	**0.010**
(B) Spatio-climatic gradient-model (*n* = 240, where *n* is species and country-specific coefficients)				
**Intercept**	**0.058 ± 0.006**	**58.612**	**9.436**	** < 0.001**
**MeanTemp**	**− 0.005 ± 0.002**	**226.256**	**− 2.474**	**0.014**
**Demog. measure (survival)**	**0.023 ± 0.008**	**218.614**	**2.915**	**0.004**
Demog. measure (survival) x MeanTemp	0.004 ± 0.003	219.208	1.326	0.186
(C) Species traits-model (*n* = 240, where *n* is species and country-specific coefficients)				
**Intercept**	**0.051 ± 0.010**	**56.275**	**4.980**	** < 0.001**
**Demog. measure (survival)**	**0.036 ± 0.013**	**215.893**	**2.876**	**0.004**
Migration	0.024 ± 0.012	43.335	1.894	0.065
Habitat	− 0.023 ± 0.016	45.151	− 1.450	0.154
**Demog. measure (survival) × Migration**	**− 0.038 ± 0.015**	**215.656**	**− 2.494**	**0.013**
Demog. measure (survival) × Habitat	0.029 ± 0.019	214.624	1.533	0.127

A, B, and C are the models to answer our three study questions from step 3 of the analyses (Fig. S1). Demographic measure (‘Demog.measure’) is adult survival or productivity, where productivity is the reference level. MeanTemp is the spatio-climatic gradient measured as a country-specific mean temperature across the years 2000–2021. Migration is the migratory strategy (long-distance migration vs. short-distance migration or resident species), where long-distance migration is the reference level. Habitat is the breeding habitat (forest habitat vs. reed habitats), where forest habitat is the reference level. Bolded values are significant (*P* < 0.05)

### The role of the spatio-climatic gradient

Model B—The mean temperature of a country (i.e., spatio-climatic gradient) did not have a significant interaction with the demographic measure (Table 1B, Fig. 2b), whereas the importance of both demographic measures increased toward the colder countries (Table 1B, Fig. 2b).

### The role of species traits

**Fig. 3:**
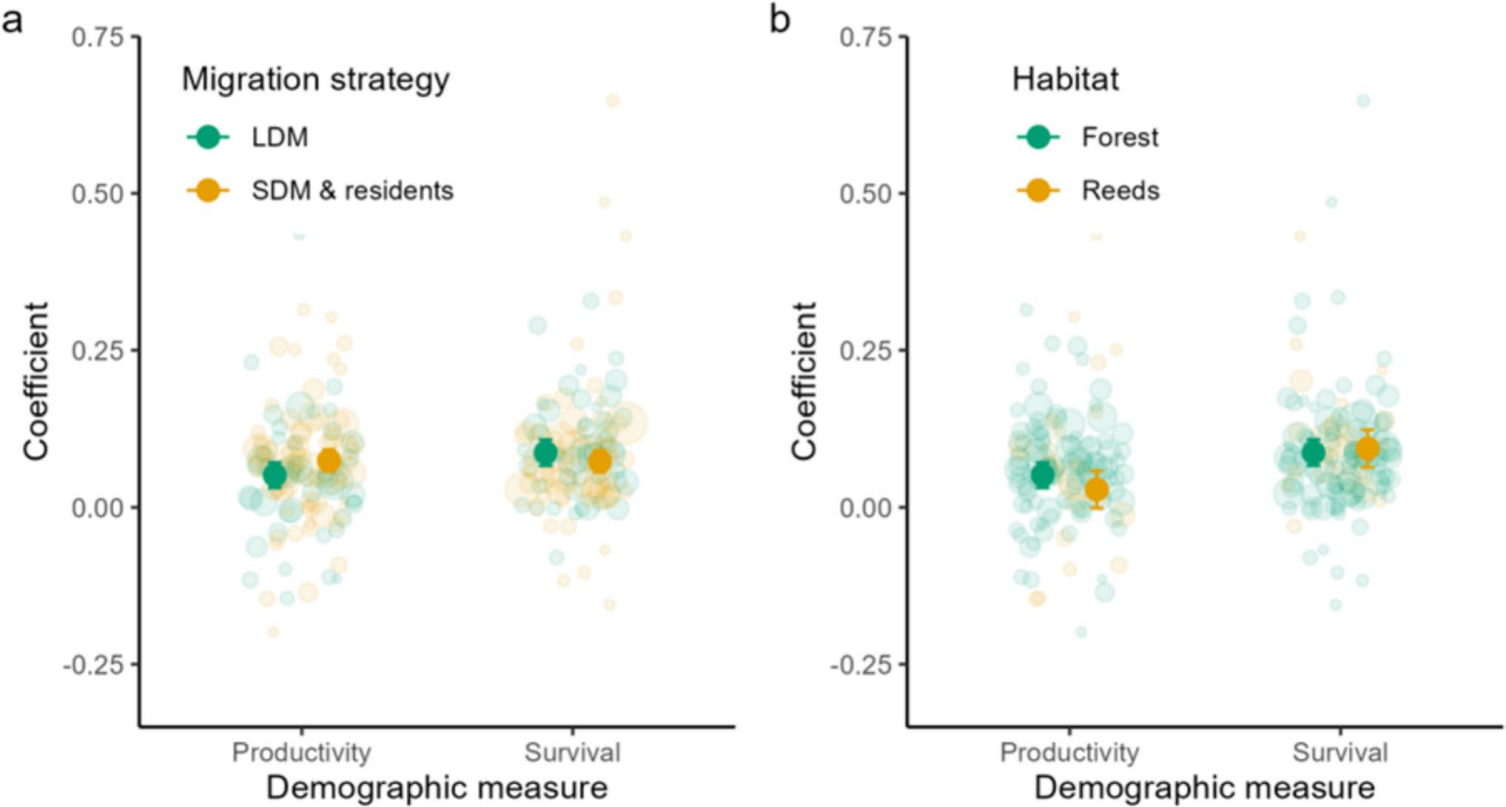
**a** The importance (coefficient) of productivity and survival on the population change of songbirds in different species traits groups: a) migratory strategy (*n* = 240, *P* = 0.013) and **b** breeding habitat (*n* = 240, *P* = 0.127). Figures are based on model C, species traits -model (Table 1), where a) the interaction between the demographic measures (productivity vs. adult survival) and the migration strategy (long-distance migrants (LDM) vs. short-distance migrants (SMD) and residents), and b) the interaction between the demographic measures (productivity vs. adult survival) and the breeding habitat (forest vs. reed habitats) is shown. Mean estimates and their confidence intervals are shown, along with the raw data points. The size of the datapoints indicates how much weight they have in the model, e.g., greater point size means smaller standard error of the estimate and thus larger weight

Model C—The relative importance of adult survival and productivity on the annual population change differed significantly between the two migratory strategies, but not between breeding habitats (Table 1C, Fig. 3). Adult survival was almost twice as important as productivity for the population change in long-distance migratory birds, while adult survival and productivity were equally important among species of short-distance and resident birds (Table 1C, Fig. 3a). In both breeding habitats (forests and reeds), adult survival appeared to have a slightly stronger effect than productivity, whereas differences between breeding habitats were not found (Table 1C, Fig. 3b).

### Sensitivity analyses

The sensitivity analyses with the three different selection criteria and the three different SE-filtering values support the robustness of our results as the results did not differ in terms of direction (positive or negative estimate) or strength of the relationships between the three selection criteria models (Supplementary material Tables S6 and S7). The spatial autocorrelation test did not find any significant spatial autocorrelation in the model residuals (Supplementary material Table S8). The inclusion of the phylogeny in PGLMMs did not change the modeling results as compared to the LMMs and revealed that the modeled relationships were not dependent on the relatedness of the species (Supplementary Material Table S9). In addition, we did not detect a phylogenetic signal in the model residuals (very low variance explained by phylogenetic covariance, see Supplementary Material Table S9).

## Discussion

Using long-term, multispecies, and continental-scale data, our study reveals that adult survival was more important than productivity for short-term annual population change of European songbirds. The roles of these demographic measures explaining annual population change did not differ along the spatio-climatic gradient. However, both adult survival and productivity were found to be more important in colder climates. We also show that adult survival had an even stronger impact on population change than productivity in long-distance migratory birds compared to short-distance migrants, whereas the role of the demographic measures did not differ between breeding habitat groups.

### The role of adult survival and productivity

In our results, both adult survival and productivity were positively associated with annual population change, yet adult survival had a stronger effect, which shows that there are differences in the roles of the two demographic parameters. As the adult survival and productivity estimates were scaled and in our final models, we compare the importance of those estimates; hence, these values cannot be directly interpreted as biologically applicable values. However, in relative terms, our results show that adult survival explained around 30% more variation in annual population change than productivity. While our data only included adult survival, results might be even more pronounced when considering also the survival of young first-year birds as survival of young is typically lower than adult survival in songbirds (e.g., Bonney et al. 2004). In addition, our measure of productivity was based on the number of young birds captured during summer, which does not tell how many will survive in the coming winter and be thereafter recruited to the breeding population. The first-winter survival of young birds is much more difficult to study compared to adults due to the low site fidelity of young birds. However, the same factors may affect the survival of both adults and young birds in the non-breeding grounds, and thus our adult survival estimates may also reflect the currently unknown survival rates of young birds. This could emphasize the role of survival overall compared to productivity. Future studies could try to investigate the importance of the role of survival of both adult and young birds, as well as the role of productivity for annual population change, from those species where the survival estimates for young birds are possible to estimate. Furthermore, we wish to acknowledge that the role of survival and productivity for population growth rate may not be the same throughout the study period, but their relative importance may change annually. Such inter-annual variation of demographic drivers would require more detailed investigations than in our study; however, earlier studies have already shown that survival is more strongly linked to annual variation in species abundance, whereas productivity is driving the trends over time (Robinson et al. 2014; Morrison et al. 2016).

Typically, adult survival has been considered more important than productivity for large-sized, long-lived bird species as it takes several years for them to mature, and their annual productivity is low (e.g., Bonney et al. 2004). Yet, our results indicate that even for small-sized, relatively short-lived passerines, which typically live only for a couple of years on average (e.g., Valkama et al. 2014), survival is more important than productivity in explaining the annual variation in population change. However, as our results show only the average situation among species, and there is a rather large variation in our raw data (Figs. 2 and 3), species-specific differences likely exist. Single-species case studies have shown such different roles between adult survival and productivity on population change (e.g., adult survival being more important, see Flint et al. 1998; Rangel-Salazar et al. 2008, productivity being more important, see Plard et al. 2020; Pfeiffer and Schaub 2023). Nevertheless, in addition to species-specific evidence, it is valuable to understand general patterns across species from a conservation perspective as they can be used to guide more general conservation actions for entire species groups.

In addition to the general differences in the role of adult survival and productivity on population change, we found a spatial gradient in their importance, which is consistent with earlier studies considering survival (Scholer et al. 2020) and productivity (e.g., Eglington et al. 2015; Desante et al. 2018; Morrison et al. 2022). When moving from warmer to colder climates, both adult survival and productivity became more important for the population change of songbirds. One explanation behind the greater importance of adult survival and productivity in colder regions could be due to the harsher and colder climatic conditions in the northern climates. The harsh conditions, as such, can cause increased variation in the mortality, fertility, and abundance of species. Furthermore, for the species composition, this means that in the north, most of the species are migratory (Forsman and Mönkkönen 2002), and as survival during migration is known to be lower than during other stages of the life cycle (Alerstam et al. 2003; Newton 2024), this may explain the increased importance of adult survival in the population change of northern birds.

Considering productivity, a higher annual variation in the climatic conditions can cause birds from northern populations to have a higher likelihood of breeding multiple times during years with warm springs (McDermott and DeGroote 2016; Morrison et al. 2019; Hoover and Schelsky 2020). In colder breeding years, there is an increase in nestling mortality (Leech and Crick 2007). Meller et al. (2018) showed that increased temperature during spring and early summer improves the annual productivity of both short- and long-distance migrants in the northern boreal zone. Also, it is good to note that, in general, the abiotic drivers, as for example direct impacts of climate, are more important for northern species populations, while in the south, biotic factors, such as competition between species, may be more important (Paquette and Hargreaves 2021).

### The role of species traits

Our results from the migratory strategy model show that adult survival is more important than productivity for population change of long-distance migratory birds as compared to short-distance migratory or sedentary species. This is in accordance with earlier studies as they have shown that migratory behavior is often a risk for a bird; survival during migration is known to be low, especially for long-distance migrants as they cross a wider range of habitats (Alerstam et al. 2003; Newton 2024). Besides the journey, the conditions on wintering sites can influence birds’ survival, for example, through climatic conditions, such as precipitation (Johnston et al. 2016; Rockwell et al. 2017). On the other hand, the coefficient of productivity for long-distance migrants was low compared to short-distance and resident species, whereas the difference in survival coefficients between the migratory groups was small. Therefore, it could be that the significance of adult survival for annual population change is driven more by the low importance of productivity than by the higher importance of survival. The reason behind the lower importance of productivity might be that long-distance migrants are more constrained by their migration schedule and therefore less able to adjust their breeding effort between different years (Hedenström 2007), or that long-distance migrants have lower annual fecundity and fewer broods than short-distance migrants (Böhning-Gaese et al. 2000; Bruderer and Salewski 2009).

Interestingly, the relative importance of adult survival or productivity for population change did not differ between the breeding habitats. However, our habitat classification was coarse, and the data were biased toward species that breed at least partly in forest habitats (25 out of 33 species were classified as forest breeders). Besides, previous studies have shown that, for example, some grassland breeding birds have low reproductive success through degradation in their breeding habitats (Fay et al. 2020), and Siberian jays *Perisoreus infaustus* have both reduced reproductive success and adult survival in more open than dense forests (Nystrand 2006). Therefore, with more accurate habitat classification and an extended dataset, differences may be found in how the habitat influences the role of adult survival and productivity on population change. Other species traits and environmental factors should be studied with the relative importance of the demographic measures for species population change, to further complement our current understanding and to give more instruments to halt the biodiversity decline.

## Supplementary Information

Below is the link to the electronic supplementary material.

Supplementary file1 (DOCX 345 kb)

## Data Availability

The CES ringing data were obtained from EURING, and it can be accessed through the application procedure (https://euring.org/data-and-codes/edb-application-procedure). The temperature data were downloaded from the CRY TS dataset (https://crudata.uea.ac.uk/cru/data/hrg). The trait for species' migratory strategies and breeding habitats data was downloaded from the AVONET database (https://opentraits.org/datasets/avonet.html). The phylogeny data were downloaded from the BirdTree database (https://birdtree.org/).

## Code availability

The R code and the data used in the analysis can be accessed through Zenodo: https://zenodo.org/records/13863058.
