# Supplementary material for: Adult survival has a stronger role than productivity in the annual population change of European songbirds
Source: Oecologia. 2025 Oct 11;207(11):173. doi: 10.1007/s00442-025-05810-4 (PMC12515112; doi:10.1007/s00442-025-05810-4)
Supplement: Supplementary file 1 — Supplementary file1 (DOCX 345 kb) [file 442_2025_5810_MOESM1_ESM.docx]

# 10. Supplementary material

**Table S1.** Scheme codes, their locations and their chosen years, number of sites, and number of species after exclusions. Germany’s three schemes (Hiddensee, Wilhelmshaven, and Radolfzell) were combined into one scheme, coded here as DEU. Out of Spain’s four schemes two, Aranzadi (ESP A) and Catalonia (ESP C), were kept separate. Schemes of Madrid and Seo were combined into one scheme, coded here as ESP B. The UK and Ireland were kept together as the two countries belong to the same scheme. Schemes are arranged here in alphabetical order according to their codes. When there are fewer sites in survival than productivity analysis due to the filtering, the number of sites in survival analysis is marked in brackets after the number of sites.

| **Scheme code** | **Location** | **Years** | **N sites** | **N species** |
| --- | --- | --- | --- | --- |
| CZP | Czech Republic | 2004-2021 | 41 | 13 |
| DEU | Germany | 2000-2021 | 85 (78) | 11 |
| ESP A | Spain Aranzadi | 2010-2021 | 13 | 5 |
| ESP B | Spain | 2000-2021 | 45 (31) | 8 |
| ESP C | Spain Catalonia | 2000-2010 | 31 | 8 |
| FIN | Finland | 2000-2021 | 45(43) | 11 |
| GBR & IRL | UK & Ireland | 2000-2021 | 190 (189) | 21 |
| HUN | Hungary | 2004-2021 | 37 | 13 |
| ITA | Italy | 2015-2021 | 11 | 3 |
| NLD | Netherlands | 2000-2021 | 58 | 17 |
| SWE | Sweden | 2004-2021 | 30 | 12 |
| **All** | **All** | **2000-2021** | **586 (562)** | **33** |

**Table S2.** Table of species used in the analyses, their migratory strategies, breeding habitats, number of individuals used in survival analysis, and number of individuals used in productivity analysis through all countries included in the study. For scheme and species-specific survival numbers, see Table 2a. For scheme and species-specific productivity numbers of adults, see Table 2b; of juveniles, see Table 2c. LDM = Long Distance Migrant, SDM = Short Distance Migrant. Productivity includes both the adult and the juvenile birds.

| **Scientific name** | **Migratory strategy** | **Breeding habitat** | **N survival** | **N productivity** |
| --- | --- | --- | --- | --- |
| *Acrocephalus arundinaceus* | LDM | Reed | 1076 | 1391 |
| *Acrocephalus melanopogon* | SDM/resident | Reed | 1905 | 4645 |
| *Acrocephalus palustris* | LDM | Forest | 11928 | 21400 |
| *Acrocephalus schoenobaenus* | LDM | Reed | 37468 | 100043 |
| *Acrocephalus scirpaceus* | LDM | Reed | 36040 | 89165 |
| *Aegithalos caudatus* | SDM/resident | Forest | 5741 | 20201 |
| *Certhia familiaris* | SDM/resident | Forest | 146 | 503 |
| *Cettia cetti* | SDM/resident | Reed | 3858 | 11393 |
| *Cyanistes caeruleus* | SDM/resident | Forest | 20710 | 84874 |
| *Emberiza citrinella* | SDM/resident | Forest | 285 | 555 |
| *Emberiza schoeniclus* | SDM/resident | Reed | 16813 | 38769 |
| *Erithacus rubecula* | SDM/resident | Forest | 16406 | 65970 |
| *Ficedula hypoleuca* | LDM | Forest | 1044 | 1802 |
| *Fringilla coelebs* | SDM/resident | Forest | 5414 | 13300 |
| *Hippolais polyglotta* | LDM | Forest | 3156 | 4641 |
| *Locustella luscinioides* | LDM | Reed | 1942 | 4417 |
| *Luscinia megarhynchos* | LDM | Forest | 5267 | 9515 |
| *Luscinia svecica* | LDM | Forest | 2669 | 3665 |
| *Parus major* | SDM/resident | Forest | 23127 | 86712 |
| *Passer montanus* | SDM/resident | Forest | 694 | 2062 |
| *Phylloscopus collybita* | LDM | Forest | 28206 | 102562 |
| *Phylloscopus trochilus* | LDM | Forest | 36374 | 103211 |
| *Prunella modularis* | SDM/resident | Forest | 11538 | 35523 |
| *Pyrrhula pyrrhula* | SDM/resident | Forest | 5935 | 12871 |
| *Sylvia atricapilla* | SDM/resident | Forest | 71956 | 178462 |
| *Sylvia borin* | LDM | Forest | 14577 | 30782 |
| *Sylvia communis* | LDM | Forest | 13866 | 38979 |
| *Sylvia curruca* | LDM | Forest | 3136 | 5986 |
| *Sylvia melanocephala* | SDM/resident | Bushes | 1516 | 4925 |
| *Troglodytes troglodytes* | SDM/resident | Forest | 14400 | 50499 |
| *Turdus iliacus* | SDM/resident | Forest | 510 | 1256 |
| *Turdus merula* | SDM/resident | Forest | 25333 | 59657 |
| *Turdus philomelos* | SDM/resident | Forest | 4191 | 9054 |
| **All** |  |  | **427227** | **1198793** |

**Table S3.** Number of birds in the survival analysis per species and scheme. Numbers are the first captures of individual birds; capture histories are not included in the numbers. The three schemes of Spain are separated with letters A, B and C. CZE = Czechia, DEU = Germany, ESP A = Spain Aranzadi, ESP B = Spain, ESP C = Spain Catalonia, FIN = Finland, GBR = Britain, IRL = Republic of Ireland, HUN = Hungary, ITA = Italy, NLD = Netherlands, SWE = Sweden. The order of the species follows that of Table S2, which can be used as a key to understanding the abbreviations of the species names.

| **Species** | **CZE** | **DEU** | **ESP A** | **ESP B** | **ESP C** | **FIN** | **GBR & IRL** | **HUN** | **ITA** | **NLD** | **SWE** | **All** |
| --- | --- | --- | --- | --- | --- | --- | --- | --- | --- | --- | --- | --- |
| *A. aru* |  |  |  |  |  |  |  | 1076 |  |  |  | **1076** |
| *A. mel* |  |  |  |  |  |  |  | 1905 |  |  |  | **1905** |
| *A. pal* | 2831 | 4341 |  |  |  |  |  |  |  | 4756 |  | **11928** |
| *A. sch* | 5919 |  |  |  |  | 7008 | 15570 |  |  | 8181 | 790 | **37468** |
| *A. sci* |  |  | 2857 |  |  | 856 | 30059 |  |  |  | 2268 | **36040** |
| *A. cau* |  |  |  |  |  |  | 5741 |  |  |  |  | **5741** |
| *C. fam* |  |  |  |  |  |  | 146 |  |  |  |  | **146** |
| *C. cet* |  |  | 629 | 1784 | 911 |  | 534 |  |  |  |  | **3858** |
| *C. cae* | 1770 | 1499 |  | 1262 |  | 1435 | 10921 |  |  | 2918 | 905 | **20710** |
| *E. cit* | 285 |  |  |  |  |  |  |  |  |  |  | **285** |
| *E. sch* | 1936 | 1576 |  |  |  | 2550 | 3378 | 923 |  | 5161 | 1289 | **16813** |
| *E. rub* |  | 1125 |  | 1515 | 1288 | 1883 | 6751 | 1247 |  | 2597 |  | **16406** |
| *F. hyp* |  |  |  |  |  | 1044 |  |  |  |  |  | **1044** |
| *F. coe* |  |  |  |  |  |  | 4432 |  |  |  | 982 | **5414** |
| *H. pol* |  |  | 796 | 1023 | 1337 |  |  |  |  |  |  | **3156** |
| *L. lus* |  |  |  |  |  |  |  | 1942 |  |  |  | **1942** |
| *L. meg* |  |  |  | 2268 | 2268 |  |  | 271 | 460 |  |  | **5267** |
| *L. sve* | 439 |  |  |  |  |  |  |  |  | 2230 |  | **2669** |
| *P. maj* | 1583 | 3351 |  |  | 763 | 1856 | 7567 | 2045 |  | 4574 | 1388 | **23127** |
| *P. mon* |  |  |  |  |  |  |  | 694 |  |  |  | **694** |
| *P. col* | 3039 | 3954 |  |  |  |  | 10343 | 849 |  | 10021 |  | **28206** |
| *P. tro* |  |  |  |  |  | 6377 | 14491 |  |  | 11369 | 4137 | **36374** |
| *P. mod* |  | 687 |  |  |  |  | 9055 |  |  | 1796 |  | **11538** |
| *P. pyr* |  |  |  |  |  |  | 5935 |  |  |  |  | **5935** |
| *S. atr* | 5759 | 12026 | 1354 | 6265 | 2063 |  | 21389 | 8368 | 1230 | 11988 | 1514 | **71956** |
| *S. bor* | 926 | 2357 |  |  |  | 1906 | 3453 |  |  | 5038 | 897 | **14577** |
| *S. com* | 831 |  |  |  |  | 1827 | 6963 |  |  | 3344 | 901 | **13866** |
| *S. cur* | 835 |  |  |  |  |  | 971 | 485 |  | 845 |  | **3136** |
| *S. mel* |  |  |  | 614 | 902 |  |  |  |  |  |  | **1516** |
| *T. tro* |  |  |  |  |  |  | 10639 |  |  | 3761 |  | **14400** |
| *T. ili* |  |  |  |  |  | 510 |  |  |  |  |  | **510** |
| *T. mer* | 1336 | 2566 | 503 | 2188 | 2251 |  | 10623 | 1665 | 455 | 3238 | 508 | **25333** |
| *T. phi* |  | 901 |  |  |  |  | 1748 | 572 |  | 970 |  | **4191** |
| **All** | **27489** | **34383** | **6139** | **16919** | **11783** | **27252** | **180709** | **22042** | **2145** | **82787** | **15579** | **427227** |

**Table S4.** Number of adult birds in the adult abundance and productivity analysis per species and per scheme. As both are counted per year, numbers represent the total number of adults entering the analysis; individuals may be counted twice if they are captured in different years. The three schemes of Spain are separated with letters A, B and C. CZE = Czechia, DEU = Germany, ESP A = Spain Aranzadi, ESP B = Spain, ESP C = Spain Catalonia, FIN = Finland, GBR = Britain, IRL = Republic of Ireland, HUN = Hungary, ITA = Italy, NLD = Netherlands, SWE = Sweden, ad = adult. The order of the species follows that of Table S2, which can be used as a key to understanding the abbreviations of the species names.

| **Species** | **CZE ad** | **DEU ad** | **ESP A ad** | **ESP B ad** | **ESPC ad** | **FIN ad** | **GBR & IRL ad** | **HUN ad** | **ITA ad** | **NLD ad** | **SWE ad** | **All** |
| --- | --- | --- | --- | --- | --- | --- | --- | --- | --- | --- | --- | --- |
| *A. aru* |  |  |  |  |  |  |  | 1121 |  |  |  | **1121** |
| *A. mel* |  |  |  |  |  |  |  | 2049 |  |  |  | **2049** |
| *A. pal* | 3050 | 4729 |  |  |  |  |  |  |  | 5018 |  | **12797** |
| *A. sch* | 6591 |  |  |  |  | 7576 | 17579 |  |  | 9035 | 854 | **41635** |
| *A. sci* |  |  | 3228 |  |  | 936 | 36440 |  |  |  | 2397 | **43001** |
| *A. cau* |  |  |  |  |  |  | 6732 |  |  |  |  | **6732** |
| *C. fam* |  |  |  |  |  |  | 186 |  |  |  |  | **186** |
| *C. cet* |  |  | 493 | 2336 | 1137 |  | 788 |  |  |  |  | **4754** |
| *C. cae* | 1992 | 1703 |  | 1357 |  | 1629 | 12607 |  |  | 3275 | 1063 | **23626** |
| *E. cit* | 336 |  |  |  |  |  |  |  |  |  |  | **336** |
| *E. sch* | 2127 | 1712 |  |  |  | 2996 | 3977 | 990 |  | 5824 | 1460 | **19086** |
| *E. rub* |  | 1208 |  | 1690 | 1416 | 1900 | 7993 | 1310 |  | 2743 |  | **18260** |
| *F. hyp* |  |  |  |  |  | 1141 |  |  |  |  |  | **1141** |
| *F. coe* |  |  |  |  |  |  | 5261 |  |  |  | 1062 | **6323** |
| *H. pol* |  |  | 866 | 1152 | 1522 |  |  |  |  |  |  | **3540** |
| *L. lus* |  |  |  |  |  |  |  | 2033 |  |  |  | **2033** |
| *L. meg* |  |  |  | 2748 | 2743 |  |  | 324 | 545 |  |  | **6360** |
| *L. sve* | 529 |  |  |  |  |  |  |  |  | 2677 |  | **3206** |
| *P. maj* | 1773 | 3814 |  |  | 851 | 2148 | 8875 | 2252 |  | 5234 | 1597 | **26544** |
| *P. mon* |  |  |  |  |  |  |  | 757 |  |  |  | **757** |
| *P. col* | 3332 | 4355 |  |  |  |  | 11417 | 910 |  | 10889 |  | **30903** |
| *P. tro* |  |  |  |  |  | 6784 | 16877 |  |  | 12949 | 4617 | **41227** |
| *P. mod* |  | 865 |  |  |  |  | 12185 |  |  | 2170 |  | **15220** |
| *P. pyr* |  |  |  |  |  |  | 6887 |  |  |  |  | **6887** |
| *S. atr* | 6266 | 13134 | 1561 | 5983 | 2331 |  | 23315 | 9115 | 1353 | 12680 | 1575 | **77313** |
| *S. bor* | 1032 | 2731 |  |  |  | 2002 | 4156 |  |  | 5950 | 1021 | **16892** |
| *S. com* | 973 |  |  |  |  | 1946 | 7945 |  |  | 3663 | 1045 | **15572** |
| *S. cur* | 997 |  |  |  |  |  | 1064 | 575 |  | 906 |  | **3542** |
| *S. mel* |  |  |  | 657 | 1039 |  |  |  |  |  |  | **1696** |
| *T. tro* |  |  |  |  |  |  | 12183 |  |  | 4298 |  | **16481** |
| *T. ili* |  |  |  |  |  | 559 |  |  |  |  |  | **559** |
| *T. mer* | 1600 | 3144 | 605 | 2508 | 2749 |  | 13788 | 1930 | 518 | 4013 | 591 | **31446** |
| *T. phi* |  | 988 |  |  |  |  | 2049 | 622 |  | 1086 |  | **4745** |
| **All** | **30598** | **38383** | **6753** | **18431** | **13788** | **29617** | **212304** | **23988** | **2416** | **92410** | **17282** | **485970** |

**Table S5.** Number of juvenile birds in the productivity analysis per species and scheme. As passerines are counted as adult in their second year, numbers represent the first captures of birds in the year they are born. The three schemes of Spain are separated with letters A, B and C. CZE = Czechia, DEU = Germany, ESP A = Spain Aranzadi, ESP B = Spain, ESP C = Spain Catalonia, FIN = Finland, GBR = Britain, IRL = Republic of Ireland, HUN = Hungary, ITA = Italy, NLD = Netherlands, SWE = Sweden, j = juvenile. The order of the species follows that of Table S2, which can be used as a key to understanding the abbreviations of the species names.

| **Species** | **CZE j** | **DEU j** | **ESP A j** | **ESP B j** | **ESP C j** | **FIN j** | **GBR & IRL j** | **HUN j** | **ITA j** | **NLD j** | **SWE j** | **All** |
| --- | --- | --- | --- | --- | --- | --- | --- | --- | --- | --- | --- | --- |
| *A. aru* |  |  |  |  |  |  |  | 270 |  |  |  | **270** |
| *A. mel* |  |  |  |  |  |  |  | 2596 |  |  |  | **2596** |
| *A. pal* | 2266 | 3130 |  |  |  |  |  |  |  | 3207 |  | **8603** |
| *A. sch* | 5434 |  |  |  |  | 10591 | 24471 |  |  | 16344 | 1568 | **58408** |
| *A. sci* |  |  | 1490 |  |  | 1809 | 40145 |  |  |  | 2720 | **46164** |
| *A. cau* |  |  |  |  |  |  | 13469 |  |  |  |  | **13469** |
| *C. fam* |  |  |  |  |  |  | 317 |  |  |  |  | **317** |
| *C. cet* |  |  | 1060 | 2571 | 1979 |  | 1029 |  |  |  |  | **6639** |
| *C. cae* | 4140 | 3942 |  | 2452 |  | 4567 | 34819 |  |  | 9238 | 2080 | **61238** |
| *E. cit* | 219 |  |  |  |  |  |  |  |  |  |  | **219** |
| *E. sch* | 1424 | 2545 |  |  |  | 3201 | 3411 | 920 |  | 7064 | 1118 | **19683** |
| *E. rub* |  | 3267 |  | 2297 | 2865 | 7820 | 24608 | 2431 |  | 4435 |  | **47723** |
| *F. hyp* |  |  |  |  |  | 661 |  |  |  |  |  | **661** |
| *F. coe* |  |  |  |  |  |  | 6300 |  |  |  | 677 | **6977** |
| *H. pol* |  |  | 423 | 278 | 400 |  |  |  |  |  |  | **1101** |
| *L. lus* |  |  |  |  |  |  |  | 2384 |  |  |  | **2384** |
| *L. meg* |  |  |  | 1062 | 1414 |  |  | 125 | 554 |  |  | **3155** |
| *L. sve* | 459 |  |  |  |  |  |  |  |  |  |  | **459** |
| *P. maj* | 3669 | 8817 |  |  | 1455 | 4395 | 21677 | 4593 |  | 12549 | 3013 | **60168** |
| *P. mon* |  |  |  |  |  |  |  | 1305 |  |  |  | **1305** |
| *P. col* | 5086 | 7476 |  |  |  |  | 40899 | 1332 |  | 16866 |  | **71659** |
| *P. tro* |  |  |  |  |  | 14324 | 29552 |  |  | 12671 | 5437 | **61984** |
| *P. mod* |  | 737 |  |  |  |  | 17411 |  |  | 2155 |  | **20303** |
| *P. pyr* |  |  |  |  |  |  | 5984 |  |  |  |  | **5984** |
| *S. atr* | 6227 | 17955 | 1926 | 3445 | 2827 |  | 47641 | 5870 | 2583 | 10904 | 1771 | **101149** |
| *S. bor* | 537 | 2568 |  |  |  | 2465 | 3929 |  |  | 3807 | 584 | **13890** |
| *S. com* | 702 |  |  |  |  | 2904 | 15579 |  |  | 3317 | 905 | **23407** |
| *S. cur* | 431 |  |  |  |  |  | 1237 | 255 |  | 521 |  | **2444** |
| *S. mel* |  |  |  | 910 | 2319 |  |  |  |  |  |  | **3229** |
| *T. tro* |  |  |  |  |  |  | 28345 |  |  | 5673 |  | **34018** |
| *T. ili* |  |  |  |  |  | 697 |  |  |  |  |  | **697** |
| *T. mer* | 1493 | 2925 | 602 | 2589 | 3137 |  | 11425 | 1376 | 902 | 3178 | 584 | **28211** |
| *T. phi* |  | 1155 |  |  |  |  | 1896 | 521 |  | 737 |  | **4309** |
| **All** | **32087** | **54517** | **5501** | **15604** | **16396** | **53434** | **374144** | **23978** | **4039** | **112666** | **20457** | **712823** |


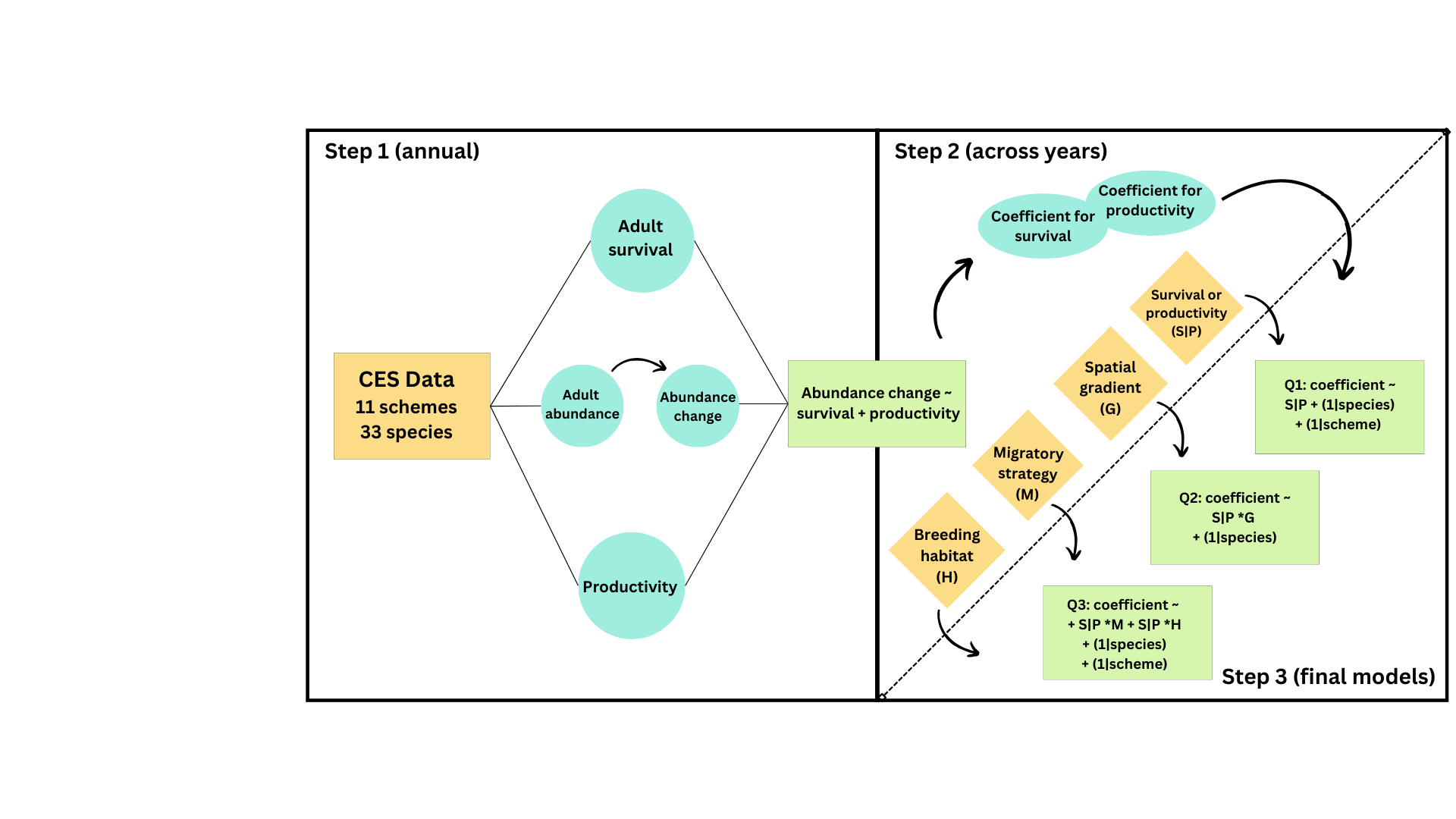


**Figure S1.** Workflow of the analysis. From CES data with 11 schemes and 33 species, we estimated adult survival, productivity and adult abundance by species and scheme (step 1). Using these estimated values, we ran a model that produced two coefficients, one for the role of adult survival and one for productivity, telling how much adult abundance changes are influenced by adult survival or productivity (step 2). The last step (step 3) was to answer our study questions by using these two coefficients from step 2. Q1: which demographic measure is more important across all species and schemes for annual population change, Q2: how does the spatio-climatic gradient influence this connection, Q3: how do species traits (migratory strategy, and breeding habitat) affect the role of adult survival or productivity in explaining annual population change?


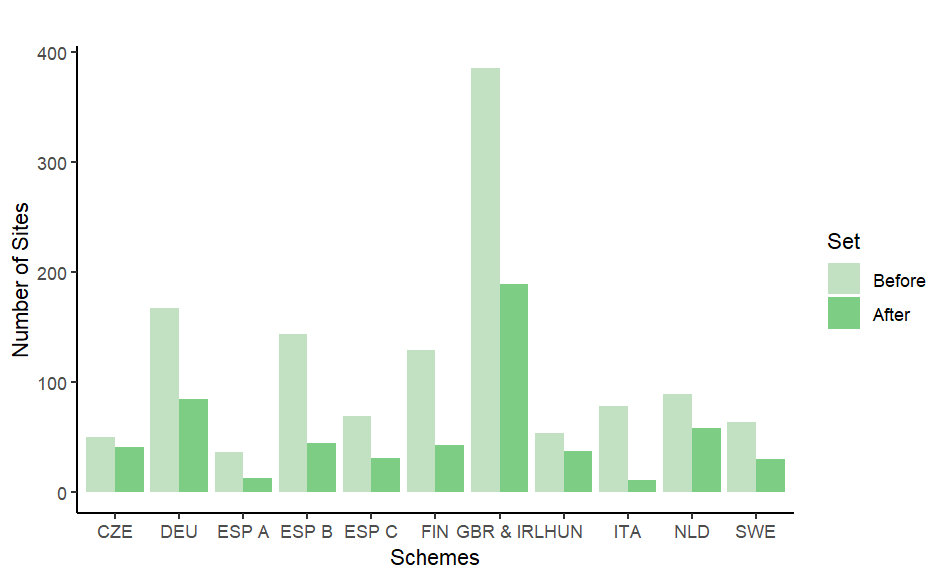


**Figure S2.** Number of sites per scheme in productivity analysis before (light green) and after (dark green) our filtering. The three schemes of Spain are separated with letters A, B and C. CZE = Czechia, DEU = Germany, ESP A = Spain Aranzadi, ESP B = Spain, ESP C = Spain Catalonia, FIN = Finland, GBR = Britain, IRL = Republic of Ireland, HUN = Hungary, ITA = Italy, NLD = Netherlands, SWE = Sweden.


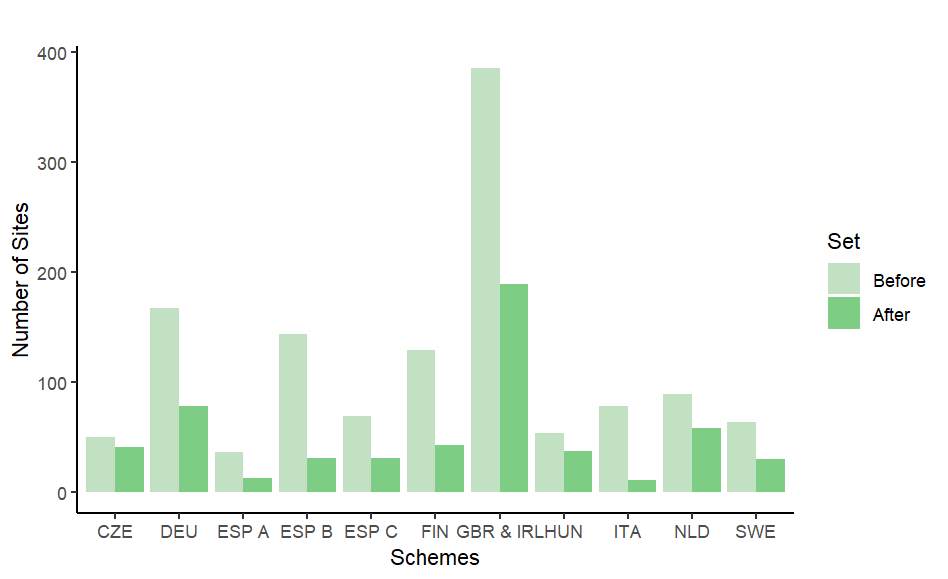


**Figure S3.** Number of sites per scheme in survival analysis before (light green) and after (dark green) our filtering. The three schemes of Spain are separated with letters A, B and C. CZE = Czechia, DEU = Germany, ESP A = Spain Aranzadi, ESP B = Spain, ESP C = Spain Catalonia, FIN = Finland, GBR = Britain, IRL = Republic of Ireland, HUN = Hungary, ITA = Italy, NLD = Netherlands, SWE = Sweden.


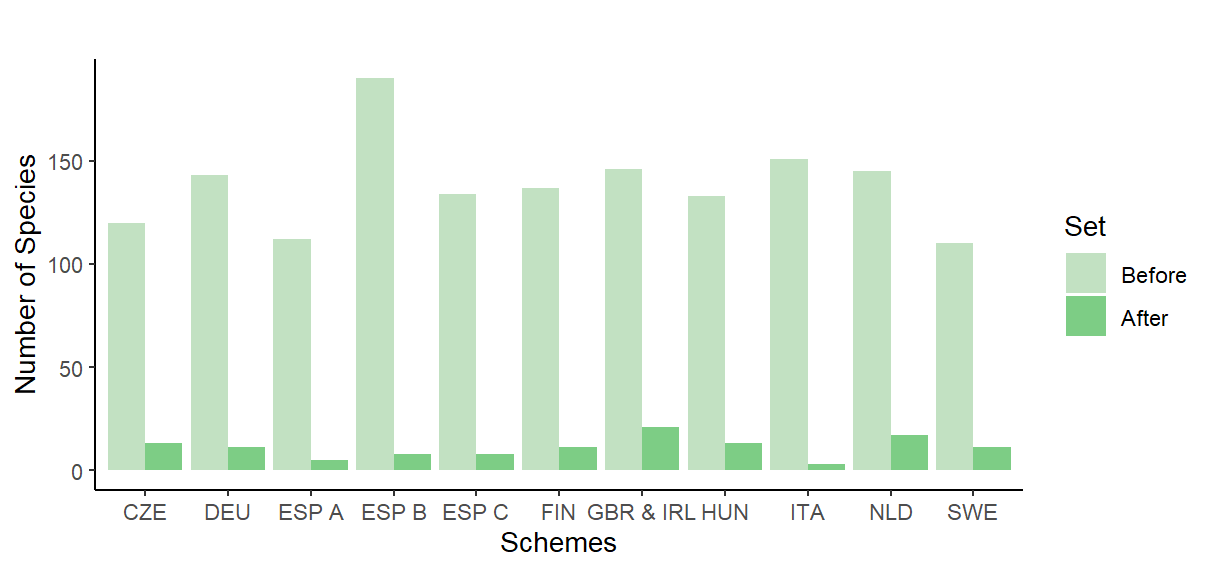


**Figure S4.** The number of species per scheme before (light green) and after (dark green) our filtering. The three schemes of Spain are separated with letters A, B and C. CZE = Czechia, DEU = Germany, ESP A = Spain Aranzadi, ESP B = Spain, ESP C = Spain Catalonia, FIN = Finland, GBR = Britain, IRL = Republic of Ireland, HUN = Hungary, ITA = Italy, NLD = Netherlands, SWE = Sweden.


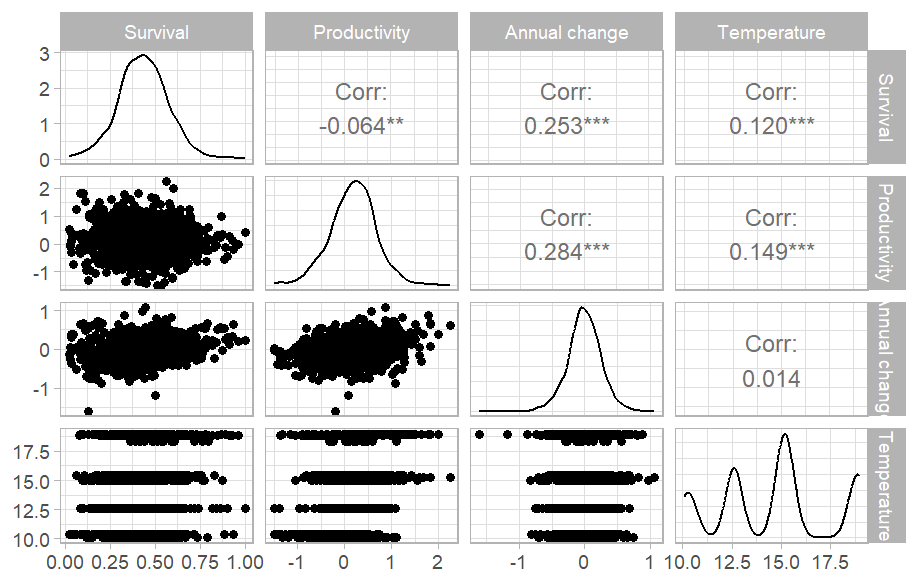


**Figure S5.** Pairwise Pearson correlation between all tested continuous variables, showing no strong collinearity (i.e. r < 0.6). Survival, productivity, and annual change are the annual values per species and scheme (i.e. obtained from cesr in step 1), whereas temperature is the average temperature per scheme across the years.

**Table S6.** Results from the first sensitivity analysis of species selection criteria. A, B, and C are the models to answer our three study questions. Demographic measure (‘Demog.measure’) is survival or productivity, where productivity is the reference level. MeanTemp is the spatio-climatic gradient measured as a country-specific mean temperature across the years 2000–2021. Migration refers to migratory strategy (long-distance migration vs. short-distance and resident species), where long-distance migration is the reference level. Habitat is breeding habitat (forest habitat vs. reed habitats), where forest habitat is the reference level. Bolded values are significant. When there are fewer sites in survival than productivity analysis due to the filtering, the number of sites in survival analysis is marked in brackets after the number of sites.

|  | **Chosen criteria** | **Strict criteria** | **Loose criteria** |
| --- | --- | --- | --- |
| **Selection criteria** | *2/3 of surveys conducted, 2 adults + 2 juveniles, 50 captures,*  *(n = 240, where n is species and country-specific coefficients)* | *4/5 of surveys conducted, 3 adults + 3 juveniles, 100 captures,*  *(n = 134, where n is species and country-specific coefficients)* | *1/2 of surveys conducted, 1 adults + 1 juveniles, 30 captures,*  *(n = 310, where n is species and country-specific coefficients)* |
| **Number of species** | 33 | 25 | 42 |
| **Number of sites** | 583 (562) | 475 (460) | 656 (630) |
| **Number of schemes** | 11 | 10 | 12 |
|  |  |  |  |
| **Parameter** | **Estimate ± SE** | **Estimate ± SE** | **Estimate ± SE** |
| 1. *Survival vs. productivity -model* | | | |
| Intercept | **0.061 ± 0.007** | **0.058 ± 0.007** | **0.061 ± 0.006** |
| Demog. measure (survival) | **0.019 ± 0.007** | **0.019 ± 0.008** | 0.010 ± 0.006 |
| Parameter | **Estimate ± SE** | **Estimate ± SE** | **Estimate ± SE** |
| 1. *Spatio-climatic gradient -model* | | | |
| Intercept | **0.058 ± 0.006** | **0.056 ± 0.007** | **0.060 ± 0.006** |
| MeanTemp | **-0.005 ± 0.002** | **-0.006 ± 0.003** | **­-0.006 ± 0.002** |
| Demog. measure (survival) | **0.023 ± 0.008** | **0.022 ± 0.009** | **0.014 ± 0.006** |
| Demog. measure (survival) x MeanTemp | 0.004 ± 0.003 | 0.004 ± 0.004 | **0.005 ± 0.002** |
| Parameter | **Estimate ± SE** | **Estimate ± SE** | **Estimate ± SE** |
| 1. *Species traits -model* | | |  |
| Intercept | **0.051 ± 0.010** | **0.049 ± 0.011** | **0.055 ± 0.010** |
| Demog. measure (survival) | **0.036 ± 0.013** | **0.036 ± 0.014** | **0.021 ± 0.011** |
| Migration | 0.024 ± 0.012 | 0.024 ± 0.014 | 0.013 ± 0.012 |
| Habitat | -0.023 ± 0.016 | -0.018 ± 0.017 | -0.009 ± 0.016 |
| Demog. measure (survival) x Migration | **-0.038 ± 0.015** | **-0.039 ± 0.017** | **-0.026 ± 0.013** |
| Demog. measure (survival) x Habitat | 0.029 ± 0.019 | 0.029 ± 0.021 | 0.021 ± 0.017 |

**Table S7.** Results from the second sensitivity analysis of survival estimates SE -filtering criteria. A, B, and C are the models to answer our three study questions. Demographic measure (‘Demog.measure’) is survival or productivity, where productivity is the reference level. MeanTemp is the spatio-climatic gradient measured as a country-specific mean temperature across the years 2000–2021. Migration is the migratory strategy (long-distance migration vs. short-distance and resident species), where long-distance migration is the reference level. Habitat is breeding habitat (forest habitat vs. reed habitats), where forest habitat is the reference level. Bolded values are significant.

|  | **Chosen filter** | **Strict filter** | **Loose filter** |
| --- | --- | --- | --- |
| **Selection criteria** | *Scheme and species-specific survival estimate years where SE > 0.01 and < 0.25,*  *(n = 240, where n is species and country-specific coefficients)* | *Scheme and species-specific survival estimate years where SE > 0.01 and < 0.20,*  *(n = 230, where n is species and country-specific coefficients)* | *Scheme and species-specific survival estimate years where SE > 0.01 and < 0.30,*  *(n = 242, where n is species and country-specific coefficients)* |
| **Number of species** | 33 | 31 | 33 |
| **Number of schemes** | 11 | 11 | 11 |
|  |  |  |  |
| **Parameter** | **Estimate ± SE** | **Estimate ± SE** | **Estimate ± SE** |
| 1. *Survival vs. productivity -model* | | | |
| Intercept | **0.061 ± 0.007** | **0.061 ± 0.007** | **0.061 ± 0.006** |
| Demog. measure (survival) | **0.019 ± 0.007** | **0.016 ± 0.007** | **0.018 ± 0.007** |
| Parameter | **Estimate ± SE** | **Estimate ± SE** | **Estimate ± SE** |
| 1. *Spatio-climatic gradient -model* | | | |
| Intercept | **0.058 ± 0.006** | **0.059 ± 0.006** | **0.057 ± 0.006** |
| MeanTemp | **-0.005 ± 0.002** | **-0.006 ± 0.002** | **­-0.006 ± 0.002** |
| Demog. measure (survival) | **0.023 ± 0.008** | **0.018 ± 0.008** | **0.024 ± 0.008** |
| Demog. measure (survival) x MeanTemp | 0.004 ± 0.003 | 0.003± 0.003 | **0.006 ± 0.003** |
| Parameter | **Estimate ± SE** | **Estimate ± SE** | **Estimate ± SE** |
| 1. *Species traits -model* | | |  |
| Intercept | **0.051 ± 0.010** | **0.054 ± 0.011** | **0.051 ± 0.010** |
| Demog. measure (survival) | **0.036 ± 0.013** | **0.026 ± 0.013** | **0.034 ± 0.012** |
| Migration | 0.024 ± 0.013 | 0.020 ± 0.013 | 0.024 ± 0.012 |
| Habitat | -0.023 ± 0.016 | -0.023 ± 0.016 | -0.024 ± 0.016 |
| Demog. measure (survival) x Migration | **-0.038 ± 0.015** | -0.029 ± 0.015 | **-0.038 ± 0.015** |
| Demog. measure (survival) x Habitat | 0.030 ± 0.019 | **0.037 ± 0.019** | 0.035± 0.019 |

**Table S8.** Results from the third sensitivity analysis of the phylogenies' influence on our models. A, B, and C are the models to answer our three study questions. Demographic measure (‘Demog.measure’) is survival or productivity, where productivity is the reference level. MeanTemp is the spatio-climatic gradient measured as a country-specific mean temperature across the years 2000–2021. Migration is a migratory strategy (long-distance migration vs. short-distance and resident species), where long-distance migration is the reference level. Habitat is breeding habitat (forest habitat vs. reed habitats), where forest habitat is the reference level. Bolded values are significant. RE = Random Effect estimated variance. As PGLMM can not include weights as our LMM analysis did, we also tested an LMM analysis without weights to see its effect on the results. Last, we also report the phylogenetic signal from the model residuals.

|  | *LMM with weights* | *LMM without weights* | *PGLMM* |
| --- | --- | --- | --- |
| **Parameter** | **Estimate ± SE** | **Estimate ± SE** | **Estimate ± SE** |
| 1. *Survival vs. productivity -model (n = 240, where n is species and country-specific coefficients)* | | | |
| Intercept | **0.061 ± 0.007** | **0.068 ± 0.012** | **0.068 ± 0.012** |
| Demog. measure (survival) | **0.019 ± 0.007** | **0.029 ± 0.012** | **0.029 ± 0.012** |
| RE Species identity | 0.000 ± 0.014 | 0.002 ± 0.043 | 0.002 ± 0.048 |
| RE Species phylogeny | - | - | 0.000 ± 0.000 |
| RE Scheme | 0.000 ± 0.006 | 0.000 ± 0.000 | 0.000 ± 0.000 |
| Residual phylogenetic signal lambda = 0.000, P - value = 1 | | | |
| **Parameter** | **Estimate ± SE** | **Estimate ± SE** | **Estimate ± SE** |
| 1. *Spatio-climatic gradient -model (n = 240, where n is species and country-specific coefficients)* | | | |
| Intercept | **0.058 ± 0.006** | **0.068 ± 0.012** | **0.068 ± 0.012** |
| MeanTemp | **-0.005 ± 0.002** | 0.002 ± 0.003 | 0.002 ± 0.003 |
| Demog. measure (survival) | **0.023 ± 0.008** | **0.029 ± 0.012** | **0.029 ± 0.012** |
| Demog. measure (survival) x MeanTemp | 0.004 ± 0.003 | -0.002 ± 0.004 | -0.002 ± 0.004 |
| RE Species identity | 0.000 ± 0.012 | 0.002 ± 0.043 | 0.002 ± 0.043 |
| RE Species phylogeny | - | - | 0.000 ± 0.000 |
| Residual phylogenetic signal lambda = 0.000, P - value = 1 | | | |
| **Parameter** | **Estimate ± SE** | **Estimate ± SE** | **Estimate ± SE** |
| 1. *Species traits -model (n = 240, where n is species and country-specific coefficients)* | | | |
| Intercept | **0.051 ± 0.010** | **0.044 ± 0.021** | **0.044 ± 0.021** |
| Demog. measure (survival) | **0.036 ± 0.013** | **0.043 ± 0.021** | **0.043 ± 0.021** |
| Migration | 0.024 ± 0.013 | 0.027 ± 0.026 | 0.027 ± 0.026 |
| Habitat | -0.023 ± 0.016 | 0.040 ± 0.031 | 0.040 ± 0.032 |
| Demog. measure (survival) x Migration | **-0.038 ± 0.015** | -0.029 ± 0.025 | -0.029 ± 0.025 |
| Demog. measure (survival) x Habitat | 0.030 ± 0.019 | 0.013 ± 0.031 | 0.013 ± 0.031 |
| RE Species identity | 0.000 ± 0.015 | 0.003 ± 0.051 | 0.003 ± 0.053 |
| RE Species phylogeny | - | - | 0.000 ± 0.000 |
| RE Scheme | 0.000 ± 0.006 | 0.000 ± 0.000 | 0.000 ± 0.000 |
| Residual phylogenetic signal lambda = 0.000, P - value = 1 | | | |

**Table S9.** Results from the fourth sensitivity analysis, the spatial autocorrelation on our models using Moran’s I test from the package DHARMa. A, B, and C are the models to answer our three study questions. Demographic measure (‘Demog.measure’) is survival or productivity, where productivity is the reference level. MeanTemp is the spatio-climatic gradient measured as a country-specific mean temperature across the years 2000–2021. Migration is a migratory strategy (long-distance migration vs. other migratory strategies), where long-distance migration is the reference level. Habitat is breeding habitat forest habitat vs. reed habitats), where forest habitat is the reference level. Bolded values are significant.

|  | *Model* |
| --- | --- |
| **Parameter** | **Estimate** |
| 1. *Survival vs. productivity -model (n = 240, where n is species and country-specific coefficients)* | |
| Observed | -0.155 |
| Expected | -0.100 |
| SD | 0.073 |
| P-value | 0.777 |
| **Parameter** | **Estimate** |
| 1. *Spatio-climatic gradient -model (n = 240, where n is species and country-specific coefficients)* | |
| Observed | -0.146 |
| Expected | -0.100 |
| SD | 0.072 |
| P-value | 0.740 |
| **Parameter** | **Estimate** |
| 1. *Species traits -model (n = 240, where n is species and country* 2. *specific coefficients)* | |
| Observed | -0.132 |
| Expected | -0.100 |
| SD | 0.073 |
| P-value | 0.669 |

**Table S10.** The structures of our three base models (A, B, and C). Demographic measure (‘Demog.measure’) is adult survival (reference level: productivity). MeanTemp is the spatio-climatic gradient (country-specific, species range-centered mean temperatures across the years 2000–2021). Migration is the migratory strategy (long-distance migration vs. short-distance and resident species), where the long-distance migration is the reference level. SDM is a short-distance migrant. Habitat is breeding habitat (forest habitat vs. reed habitats), where forest habitat is the reference level. Bolded values are significant.

| **Model** | **Response variable** | **Explanatory variables** |  | **Random effects** |
| --- | --- | --- | --- | --- |
| A | Coefficient of demog. measure | Demog. measure | | Species, scheme |
| B | Coefficient of demog. measure | Demog.measure*MeanTemp | | Species |
| C | Coefficient of demog. neasure | Demog.measure*Migration + Demog.measure*Habitat | | Species, scheme |
